# Population-level data on antenatal screening for proteinuria; India, Mozambique, Nigeria, Pakistan

**DOI:** 10.2471/BLT.19.248898

**Published:** 2020-09-09

**Authors:** Laura A Magee, Sumedha Sharma, Esperança Sevene, Rahat N Qureshi, Ashalata Mallapur, Salésio E Macuácua, Shivaprasad Goudar, Mrutunjaya B Bellad, Olalekan O Adetoro, Beth A Payne, John Sotunsa, Anifa Valá, Jeffrey Bone, Andrew H Shennan, Marianne Vidler, Zulfiqar A Bhutta, Peter von Dadelszen, & the Community-Level Interventions for Pre-eclampsia Study Group

**Affiliations:** a Department of Women and Children’s Health, King’s College London, Becket House, 1 Lambeth Palace Road, SE1 7EU, London, England.; b Department of Obstetrics and Gynaecology, University of British Columbia, Vancouver, Canada.; c Centro de Investigação em Saúde da Manhiça, Universidade Eduardo Mondlane, Maputo, Mozambique.; d Centre of Excellence, Aga Khan University, Karachi, Pakistan.; e S Nijalingappa Medical College and HSK (Hanagal Shree Kumareshwar) Hospital and Research Centre, Bagalkote, India.; f KLE Academy of Higher Education and Research’s J N Medical College Belagavi, Karnataka, India.; g Department of Obstetrics and Gynaecology, Olabisi Onabanjo University, Ago Iwoye, Nigeria.; h Centre for International Child Health, University of British Columbia, Vancouver, Canada.; i Babcock University Teaching Hospital, Ilishan-Remo, Nigeria.; j Centre for Global Child Health, Hospital for Sick Children, Toronto, Canada.

## Abstract

**Objective:**

To estimate the prevalence and prognosis of proteinuria at enrolment in the 27 intervention clusters of the Community-Level Interventions for Pre-eclampsia cluster randomized trials.

**Methods:**

We identified pregnant women eligible for inclusion in the trials in their communities in four countries (2013–2017). We included women who delivered by trial end and received an intervention antenatal care visit. The intervention was a community health worker providing supplementary hypertension-oriented care, including proteinuria assessment by visual assessment of urinary dipstick at the first visit and all subsequent visits when hypertension was detected. In a multilevel regression model, we compared baseline prevalence of proteinuria (≥ 1+ or ≥ 2+) across countries. We compared the incidence of subsequent complications by baseline proteinuria.

**Findings:**

Baseline proteinuria was detected in less than 5% of eligible pregnancies in each country (India: 234/6120; Mozambique: 94/4234; Nigeria: 286/7004; Pakistan: 315/10 885), almost always with normotension (India: 225/234; Mozambique: 93/94; Nigeria: 241/286; Pakistan: 264/315). There was no consistent relationship between baseline proteinuria (either ≥ 1+ or ≥ 2+) and progression to hypertension, maternal mortality or morbidity, birth at < 37 weeks, caesarean section delivery or perinatal mortality or morbidity. If proteinuria testing were restricted to women with hypertension, we projected annual cost savings of 153 223 981 United States dollars (US$) in India, US$ 9 055 286 in Mozambique, US$ 53 181 933 in Nigeria and US$ 38 828 746 in Pakistan.

**Conclusion:**

Our findings question the recommendations to routinely evaluate proteinuria at first assessment in pregnancy. Restricting proteinuria testing to pregnant women with hypertension has the potential to save resources.

## Résumé

**Objectif:**

Estimer la prévalence et le pronostic de la protéinurie au moment de l'inscription dans l'un des 27 groupes repris dans les essais randomisés en grappes sur les interventions communautaires liées à la prééclampsie.

**Méthodes:**

Nous avons sélectionné des femmes enceintes remplissant les conditions requises pour les essais menés au sein de leur communauté dans quatre pays (2013–2017). Nous avons inclus des femmes ayant accouché au terme de l'essai et ayant fait l'objet d'une consultation prénatale avec intervention. L'intervention était menée par un professionnel de santé communautaire prodiguant des soins complémentaires en lien avec l'hypertension, dont un contrôle de la protéinurie par examen visuel d'une bandelette réactive trempée dans les urines lors de la première visite, ainsi que lors des visites qui suivent tout diagnostic d'hypertension. Nous avons employé un modèle de régression multiniveaux pour analyser la prévalence initiale de la protéinurie (≥ 1+ ou ≥ 2+) dans les différents pays. Enfin, nous avons comparé l'incidence des complications ultérieures en fonction de la protéinurie initiale.

**Résultats:**

La protéinurie initiale a été détectée chez moins de 5% des grossesses étudiées dans chaque pays (Inde: 234/6120; Mozambique: 94/4234; Nigeria: 286/7004, Pakistan: 315/10 885), et allait presque toujours de pair avec une tension artérielle normale (Inde: 225/234; Mozambique: 93/94; Nigeria: 241/286; Pakistan: 264/315). Nous n'avons identifié aucun lien concluant entre la protéinurie initiale (qu'elle soit ≥ 1+ ou ≥ 2+) et une progression vers de l'hypertension, une mortalité ou morbidité maternelle, une naissance avant 37 semaines, un accouchement par césarienne, ou encore une mortalité ou morbidité périnatale. Si les tests de dépistage de protéinurie étaient réservés aux femmes souffrant d'hypertension, les économies réalisées chaque année représenteraient 153 223 981 dollars américains en Inde, 9 055 286 dollars américains au Mozambique, 53 181 933 dollars américains au Nigeria et 38 828 746 dollars américains au Pakistan.

**Conclusion:**

Nos résultats remettent en question les recommandations selon lesquelles il convient de contrôler régulièrement la protéinurie dès le premier bilan de grossesse. Limiter les tests aux femmes enceintes souffrant d'hypertension pourrait potentiellement permettre de ménager les ressources.

## Resumen

**Objetivo:**

Estimar la prevalencia y el pronóstico de la proteinuria al momento de la inscripción en los 27 grupos de intervención de los ensayos aleatorizados del grupo de intervenciones a nivel comunitario para la preeclampsia.

**Métodos:**

Se identificaron mujeres embarazadas que cumplían los requisitos para participar en los ensayos en sus comunidades en cuatro países (2013-2017). Se incluyeron las mujeres que tuvieron un parto al final del ensayo y recibieron una visita de atención prenatal de intervención. Esta intervención consistió en un profesional sanitario de la comunidad quien prestó atención suplementaria centrada en la hipertensión, incluida la evaluación de la proteinuria a través del análisis visual de la tira reactiva de orina en la primera visita y en todas las visitas posteriores si se presentaba hipertensión. En un modelo de regresión multinivel, se comparó la prevalencia del valor basal de la proteinuria (≥ 1+ o ≥ 2+) en todos los países. Luego, se comparó la incidencia de las complicaciones posteriores en función del valor basal de la proteinuria.

**Resultados:**

El valor basal de la proteinuria se detectó en menos del 5 % de los embarazos que cumplían los requisitos en cada país (India: 234/6120; Mozambique: 94/4234; Nigeria: 286/7004, Pakistán: 315/10 885), la mayoría de las veces con una tensión arterial normal (India: 225/234; Mozambique: 93/94; Nigeria: 241/286; Pakistán: 264/315). No se observó una relación coherente entre el valor basal de la proteinuria (ya sea ≥ 1+ o ≥ 2+) y la progresión a la hipertensión, la mortalidad o morbilidad materna, el nacimiento a las < 37 semanas, el parto por cesárea o la mortalidad o morbilidad perinatal. Si la prueba de proteinuria se limitara a las mujeres con hipertensión, se calcularían ahorros anuales de 153 223 981 dólares estadounidenses (USD) en la India, 9 055 286 USD en Mozambique, 53 181 933 USD en Nigeria y 38 828 746 USD en Pakistán.

**Conclusión:**

Los resultados cuestionan las recomendaciones de evaluar la proteinuria de forma rutinaria en la primera evaluación durante el embarazo. Limitar la prueba de proteinuria a las mujeres embarazadas con hipertensión podría suponer un ahorro de recursos.

## ملخص

الغرض تقدير مدى انتشار البيلة البروتينية والتنبؤ بها عند الالتحاق بمجموعات التدخل الـ 27 للتدخلات على مستوى المجتمع للتجارب العشوائية العنقودية لمقدمات الارتعاج.

الطريقة قمنا بتحديد النساء الحوامل المؤهلات للمشاركة في التجارب في مجتمعاتهن في أربعة بلدان (الفترة من 2013 إلى 2017). قمنا بتضمين النساء اللواتي ولدن بنهاية التجربة، واستقبلن زيارة رعاية للتدخل سابقة للولادة. كان التدخل بواسطة عامل للرعاية الصحية المجتمعية يقدم رعاية تكميلية موجهة لارتفاع ضغط الدم، بما في ذلك تقييم البيلة البروتينية عن طريق التقييم البصري لمقياس البول في الزيارة الأولى وجميع الزيارات اللاحقة بعد الكشف عن ارتفاع ضغط الدم. في نموذج للتحوف متعدد المستويات، قمنا بمقارنة الانتشار الأساسي للبيلة البروتينية (≥ +1 أو ≥ +2) عبر البلدان. كما قمنا بمقارنة تكرار حدوث المضاعفات اللاحقة بواسطة البيلة البروتينية الأساسية.

النتائج تم اكتشاف بيلة بروتينية أساسية في أقل من 5% من حالات الحمل المؤهلة في كل بلد (الهند: 234/6120؛ موزامبيق: 94/4234؛ نيجيريا: 286/7004، باكستان: 315/10885)، دائمًا تقريبًا مع ضغط طبيعي (الهند: 225/234؛ موزامبيق: 93/94؛ نيجيريا: 241/286، باكستان: 264/315). لم تكن هناك علاقة متسقة بين البيلة البروتينية الأساسية (إما ≥ +1 أو ≥ 2+)، والتدهور إلى ارتفاع ضغط الدم، أو وفيات الأمهات، أو الإصابة بالمرض، أو الولادة عند أقل من 37 أسبوعًا، أو الولادة القيصرية، أو وفيات الفترة المحيطة بالولادة أو الإصابة بالمرض. إذا كان اختبار البيلة البروتينية مقصورًا على النساء اللاتي تعانين من ارتفاع ضغط الدم، فقد توقعنا أحجام توفير في التكلفة السنوية تبلغ 153223981 دولارًا أمريكيًا (بالدولار الأمريكي) في الهند، و9055286 دولارًا أمريكيًا في موزامبيق، و53181933 دولارًا أمريكيًا في نيجيريا، و38828746 دولارًا أمريكيًا في باكستان.

الاستنتاج لا تثق النتائج التي توصلنا إليها في التوصيات بتقييم البيلة البروتينية بشكل روتيني عند التقييم الأول في الحمل. إن اقتصار اختبار البيلة البروتينية على النساء الحوامل اللاتي تعانين من ارتفاع ضغط الدم، من شأنه أن يوفر في الموارد.

## 摘要

**目的:**

评估先兆子痫群体随机试验中，社区干预的 27 个干预群体中蛋白尿的患病率和预后。

**方法:**

我们在这四个国家中选取了符合其社区试验条件的孕妇（2013-2017 年）。其中包括试验结束前分娩且接受了干预产前检查的女性。由社区卫生工作人员提供侧重高血压的补充检查，包括第一次就诊以及在后续就诊期间均检测出高血压时，通过目测评估尿试纸，对蛋白尿进行评估。在多层回归模型中，我们比较了各国蛋白尿（1+ 或 2+）的患病率基线。我们通过基线蛋白尿水平比较了后续并发症的病发率。

**结果:**

每个国家只有不到 5％ 的符合试验条件的孕妇检测了基线蛋白尿水平（巴基斯坦：315/10885、莫桑比克：94/4234、尼日利亚：286/7004、印度：234/6120），其中血压几乎都正常（巴基斯坦：264/315、莫桑比克：93/94、尼日利亚：241/286、印度：225/234）。基线蛋白尿水平（1+ 或 2+）与发展为高血压、孕产妇死亡率或发病率、不足 37 周出生、剖腹产或围产期死亡率或发病率之间，不存在一致的正向关系。据估计，如果蛋白尿检测仅限于高血压孕妇，巴基斯坦每年可节省 38828746 美元，莫桑比克节省 9055286 美元，尼日利亚节省 53181933 美元，印度 153223981 美元。

**结论:**

根据我们的研究结果，我们不建议首次妊娠检查时进行常规蛋白尿评估。我们认为，仅限对高血压孕妇进行蛋白尿检测可能会节省财力。

## Резюме

**Цель:**

Оценить распространенность и прогноз протеинурии на момент включения в исследование у пациенток, вошедших по 27 кластерам вмешательств в рандомизированные исследования по кластеру преэклампсии «Меры вмешательства на уровне сообществ».

**Методы:**

Авторы определили беременных женщин, подходящих для включения в исследования в их сообществах в четырех странах (2013–2017 гг.). Рассматривались женщины, которые родили к концу исследования и к которым применялись определенные вмешательства в ходе визита с целью получения дородовой помощи. Меры вмешательства заключались в том, что местный медицинский работник оказывал дополнительную помощь, ориентированную на гипертензию, включая оценку протеинурии, с помощью визуальной оценки полоски для анализа мочи при первом посещении и во время всех последующих посещений при обнаружении гипертензии. В многоуровневой регрессионной модели авторы сравнили исходную распространенность протеинурии (≥1+ или ≥2+) в четырех странах. Авторы также сравнили частоту последующих осложнений с базовым уровнем протеинурии.

**Результаты:**

Исходная протеинурия была обнаружена менее чем у 5% подходящих беременностей в каждой стране (Индия: 234/6120; Мозамбик: 94/4234; Нигерия: 286/7004, Пакистан: 315/10 885) и почти всегда сопровождалась нормотензией (Индия: 225/234; Мозамбик: 93/94; Нигерия: 241/286; Пакистан: 264/315). Отсутствовала устойчивая взаимосвязь между исходной протеинурией (≥1+ или ≥2+) и переходом в гипертензию, материнской смертностью или заболеваемостью, рождением на сроке <37 недель, родоразрешением путем кесарева сечения, перинатальной смертностью или заболеваемостью. Если бы тестирование протеинурии ограничивалось женщинами с гипертензией, то возможно было бы спрогнозировать ежегодную экономию затрат в размере 153 223 981 доллара США (US $) в Индии, 9 055 286 долларов США в Мозамбике, 53 181 933 долларов США в Нигерии и 38 828 746 долларов США в Пакистане.

**Вывод:**

Полученные результаты ставят под сомнение рекомендации по регулярной оценке протеинурии при первой оценке беременности. Исследование на протеинурию только тех беременных женщин, у которых наблюдается гипертензия, может сэкономить ресурсы.

## Introduction

Hypertensive disorders of pregnancy are a leading cause of maternal and perinatal death and disability worldwide. As such, antenatal care is devoted in large part to the detection of pregnancy hypertension and in particular pre-eclampsia. Pre-eclampsia is the most dangerous form of pregnancy hypertension, being responsible for approximately one quarter of maternal deaths and serious near-miss morbidities.^1^

Pre-eclampsia most commonly manifests as hypertension and proteinuria, so the World Health Organization (WHO) recommends and considers essential the measurement of blood pressure and proteinuria at each antenatal care contact.^2^ While antenatal proteinuria testing for pregnancies at 20 or more weeks gestation has the potential to detect the proteinuria of pre-eclampsia, such testing at any gestational age might reveal underlying chronic kidney disease, which is itself associated with adverse outcomes.

The value of proteinuria testing at antenatal care contacts for pregnant women without high blood pressure has been questioned, however. First, WHO, in a discussion of asymptomatic bacteriuria, endorsed the widely-held view that dipstick proteinuria testing for pre-eclampsia has low diagnostic accuracy.^2^ Second, proteinuria testing may be less specific in very hot climates or during dry seasons when women may become dehydrated. Third, it is rare for women to present with proteinuria before the hypertension of pre-eclampsia.^3^ Fourth, proteinuria screening may impede progress towards group antenatal care given the need for privacy and toilet facilities. Finally, devoting resources to routine proteinuria screening has been questioned when most antenatal care contacts will not be associated with proteinuria.^4^

The Community-Level Interventions for Pre-eclampsia trials were cluster randomized controlled trials of community health worker (CHW)-based diagnosis and initial management of women with hypertension in pregnancy. The trials took place in four low- or lower-middle-income settings in India, Mozambique, Nigeria and Pakistan.^5^ Proteinuria testing was performed at baseline for all women and at all subsequent visits if elevated blood pressure were found. We report the incidence of baseline proteinuria assessed at the first visit and the relationship between baseline proteinuria and hypertension, preterm birth, caesarean section delivery, and maternal and perinatal mortality and morbidity. 

## Methods

In this exploratory secondary analysis, we included data from the 27 intervention clusters of the Community-Level Interventions for Pre-eclampsia cluster randomized controlled trials (NCT01911494).^5^ The trials comprised primary rural clusters: six clusters in Karnataka state, India (2013–2016); six clusters in Maputo and Gaza provinces, Mozambique (2014–2017); five clusters in Ogun state, Nigeria (2013–2015); and 10 clusters in Sindh province, Pakistan (2013–2016). The trials were approved by the research ethics board of the University of British Columbia as the coordinating centre (H12–03497) and within each country (MDC/IECHSR/2013–14/A, India; 219/CNBS/13, Mozambique; OOUTH/DA.326/T/1/, Nigeria; and 2590-Obs-ERC-13, Pakistan). The protocol is published^5^ and included in the authors’ data repository,^6^ along with the statistical analysis plan and STROBE (Strengthening the Reporting of Observational studies in Epidemiology) checklist.^6^

### Trial design

We enrolled women aged 12–49 years in the trials after they had confirmed their pregnancy and given informed consent. The intervention was carried out in a community setting and consisted of community engagement and a clinical assessment with initial treatments and referrals to health-care facilities provided by a CHW. The CHW was guided by a mobile health application based on the miniPIERS Pre-eclampsia Integrated Estimate of RiSk predictive model in hypertensive pregnancy. The application, running on mobile devices (tablet computers), provided step-by-step guidance on assessment and decision support for triage, transport and treatment of women with hypertension or emergency medical conditions.^7^^–^^9^

In the trial protocol, visits were recommended for women at least every 4 weeks before birth. CHWs measured the women’s blood pressure at every intervention visit in a standardized fashion using a device validated for use in pregnancy and pre-eclampsia (3AS1–2^®^ semi-automatic blood pressure monitor, Microlife, Clearwater, United States of America).^10^ The CHWs also carried out proteinuria screening for all women at the first intervention visit and at subsequent visits only if hypertension were detected. Proteinuria screening was carried out by visual assessment of urinary dipsticks. Women in control clusters received usual care, consisting of blood pressure measurement (using the device available) and proteinuria testing at each antenatal care contact, according to WHO guidelines.^2^

The primary outcome measure was a composite of maternal, fetal and newborn mortality and serious morbidity, such as eclampsia or pulmonary oedema. Maternal mortality was measured to 6 weeks and neonatal mortality to 28 days after birth. Hypertension was a systolic blood pressure ≥ 140 mmHg or a diastolic blood pressure ≥ 90 mmHg. Proteinuria was defined in two ways, as a urinary dipstick result of ≥ 1+ or ≥ 2+, according to manufacturer’s instructions.

Surveillance data were collected by a separate team, by household survey (quarterly in Pakistan and 6-monthly in Mozambique and Nigeria) or a research registry (India).^11^ In Nigeria, trial surveillance was suspended and the trial closed after the pilot phase because of challenges with data collection. In all countries in the trials the data were entered directly onto the mobile devices by the CHWs.

Data entered on mobile devices were synchronized and stored on the Research Electronic Data Capture servers. We transferred de-identified data from the trial (intervention clusters) and surveillance data (all clusters) to the University of British Columbia Community-Level Interventions for Pre-eclampsia coordinating centre. Data management protocols ensured security (encryption), tracking (user identification numbers and audit trails) and synchronization between devices within the cluster and with the server.

### Analysis

For this analysis, we included pregnancies in intervention clusters in which the woman had received at least one mobile application-guided (intervention) visit and delivered by the trial end. Women in control clusters did not receive intervention visits, by definition. We excluded pregnancies in which the woman was still on follow-up and undelivered to avoid underestimation of hypertension and adverse outcomes.

Our analyses included those pregnancies with complete information for variables of interest. We treated intervention clusters in each country as one cohort for our primary analysis comparing proteinuria prevalence at booking, that is, the first intervention visit of the study. We summarized continuous data by median and interquartile range and categorical data by number and proportion. Four-way between-country comparisons were made by *χ^2^*-test for categorical variables, or Kruskal–Wallis test for continuous variables, as appropriate. When comparisons were significant, we made pairwise comparisons by *χ^2^*-test and Wilcoxon rank sum test, as appropriate, to ascertain differences among countries.

We used logistic regression adjusted for country to compare proteinuria prevalence at the first intervention visit among countries. To explore whether proteinuria was related to pregnancy outcomes within a country, we matched controls to each woman with proteinuria according to individual characteristics: maternal age, parity, basic education, gestational age at enrolment (usually by last menstrual period), cluster and distance to facility.^12^ This analysis was not possible in Nigeria due to the absence of outcome data. We pooled data and calculated the overall odds ratios (ORs) for outcomes. We computed confidence intervals (CIs) for each outcome via bootstrapping, through 1000 iterations of the entire matching process. We did this to quantify variability in matching as there were many possible control matches for each case, and to prevent the results being dependent on which match was chosen.

We estimated the financial implications of a strategy of testing proteinuria only for women with hypertension. We estimated the number of antenatal care visits at which proteinuria testing would be avoided by using national estimates of annual number of births (India: 24 229 725; Mozambique: 1 085 797; Nigeria: 7 329 535; Pakistan: 5 945 845).^13^ We also estimated the incidence of normotension in pregnancy from population-based estimates of pregnancy hypertension from the trial data (India: 10.3%; Mozambique: 10.9%; Nigeria: 10.2%; Pakistan: 9.3%)^14^ and an eight-visit antenatal care contact model as per WHO guidelines.^2^ We calculated the cost of supplies used for proteinuria testing from the budgetary statements of the trials (2013–2017) in United States dollars (US$), inflated to 2019 US$.^15^

We made four additional sensitivity analyses. We explored whether between-country differences in proteinuria were affected by women’s baseline characteristics: age and parity (in all countries), maternal basic education (except in Nigeria) and gestational age at the first intervention visit. We also explored the effect of multiple pregnancies by excluding women with more than one pregnancy (in all countries), and the effect of antiretroviral therapy by excluding women with human immunodeficiency virus infection (HIV; in Mozambique). Finally, we estimated the financial implications of a strategy of testing proteinuria only for women with hypertension based on the former four-visit model. 

We performed analyses using R programming software, version 3.3.2 (R Foundation for Statistical Computing, Vienna, Austria).^16^ *P* < 0.05 was considered statistically significant.

## Results

**Fig. 1:**
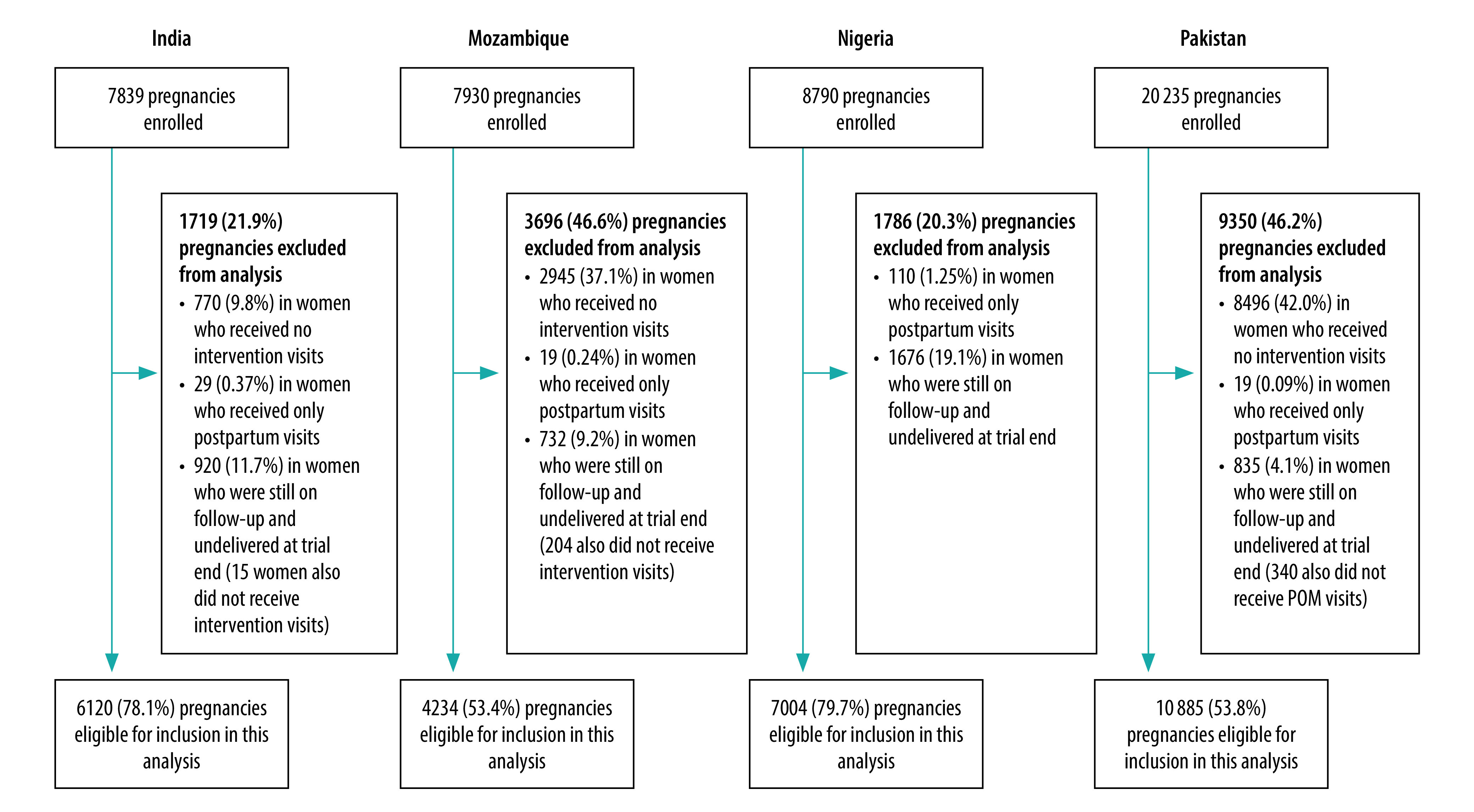
Selection of study participants in the Community-Level Interventions for Pre-eclampsia trial

Note: Data on enrolment were derived from all women who had an intervention antenatal care visit, as trial surveillance data were not available.

Of 44 794 pregnancies in the trial clusters, 12 211 (27.2%) women did not receive an intervention visit, 177 (0.4%) did not receive an intervention visit antepartum and 4163 (9.3%) were not delivered by the end of the trial. Therefore 28 243 (63.1%) pregnancies were included in this study (India: 6120; Mozambique: 4234; Nigeria: 7004; Pakistan: 10 885; Fig. 1).

The baseline characteristics of the included pregnancies differed across countries (Table 1). At enrolment in the trials, women in India and Mozambique were slightly younger than those in Pakistan and Nigeria. About one-third of women were nulliparous, except in Pakistan where the proportion was closer to one-fifth. Women in India were enrolled earlier, at the end of the first trimester, compared with early (Nigeria and Pakistan) or late (Mozambique) second trimester in the other countries. Levels of maternal education were low, particularly in Pakistan. The prevalence of HIV-positivity was 23.0% (972/4234) in Mozambique, the only country where this was measured, and the majority of HIV-positive women were on antiretroviral therapy. There were few women with multiple pregnancies, although more in Mozambique. Women in all countries delivered at a median of 39 weeks, although slightly earlier in Pakistan. The trials’ data did not include information about smoking, body mass index or prior pre-eclampsia (due to low health literacy of the mothers). In all countries, women who received an intervention visit(s) were similar at baseline to those who did not, although gestational age at enrolment in the trials was 2–4 weeks earlier (data repository);^6^ information was unavailable in Nigeria.

**Table 1:** Characteristics of women in the Community-Level Interventions for Pre-eclampsia trials who received one or more intervention visits and had delivered by end of the trial, 2013–2017

Variable	India (*n* = 6 120)	Mozambique (*n* = 4 234)	Nigeria (*n* = 7 004)	Pakistan (*n* = 10 885)	*P*^a^
Maternal age, median years (IQR)	23 (20–25)	23 (19–30)	27 (23–31)	28 (25–30)	< 0.001
Missing values (%)	0 (0.0)	146 (3.4)	10 (0.1)	22 (0.2)	NA
Nulliparous, no. (%)	2 212 (36.1)	1 280 (30.2)	2 159 (30.6)	2 476 (22.7)	< 0.001
Maternal basic education, no. (%)^b^	3 545 (57.9)	2 474 (58.4)	NA^c^	2 482 (22.8)	< 0.001
HIV-positive by maternal report, no. (%)	NA	972 (23.0)	NA^c^	NA^d^	NA
Received antiretroviral therapy for HIV, no. (% of HIV-positive)	NA	839 (86.3)	NA^c^	NA^d^	NA
Multiple pregnancies, no. (%)	53 (0.9)	105 (2.5)	NA^c^	86 (0.8)	< 0.001
Gestational age at trial enrolment, median weeks (IQR)	10.4 (7.9–14.1)	25.1 (19.5–30.9)	16.6 (13.4–18.4)	18.7 (13.6–24.6)	< 0.001
Gestational age at delivery, median weeks (IQR)	39.0 (38.0–40.0)	39.3 (37.3–41.0)	39.3 (37.4–40.7)	38.6 (36.1–40.7)	< 0.001

HIV: human immunodeficiency virus; IQR: interquartile range; NA: not applicable.

^a^ *P*-value was based on comparisons of all groups by Kruskal–Wallis test for continuous variables, and *χ^2^*-test for categorical variables, as appropriate.

^b^ Maternal basic education was defined as 8 years or more of schooling (India), achievement of grade 5 or above (Mozambique) and 5 years or more of schooling (Pakistan).

^c^ Trial surveillance data were not available for Nigeria.

^d^ Questions about HIV and antiretroviral therapy were not asked in India and Pakistan.

Notes: *n* is the total number of pregnancies included in this analysis. At intervention visits women received a clinical assessment by a community health worker guided by a mobile health application, including dipstick proteinuria assessment at the first community-level visit and all subsequent visits when hypertension was detected.

The frequency and quality of intervention antenatal visits has been previously reported.^14^ In brief, most intervention visits began 2–4 weeks after enrolment in the trials (except in Nigeria where there was a larger delay) and at < 20 weeks in most pregnancies in India, just under half in Pakistan, and a distinct minority in Mozambique and Nigeria (Table 2). This resulted in more intervention visits in India than in the other countries. Proteinuria screening was undertaken at the first intervention visit for more than 90.0% of pregnancies in each country, and at subsequent antenatal visits for more than 90.0% of pregnancies with hypertension detected, except in Nigeria where it was 80.7% (Table 2). Each country team chose a different type of proteinuria test strip, although all were assessed visually according to each manufacturer’s instructions; India and Mozambique used a multitest strip, whereas Nigeria and Pakistan used strips that had one additional test (glucose).

**Table 2:** Quality and nature of antenatal visits in the Community-Level Interventions for Pre-eclampsia trial in pregnancies in intervention clusters, 2013–2017

Variable	India (*n* = 6 120)	Mozambique (*n* = 4 234)	Nigeria (*n* = 7 004)	Pakistan (*n* = 10 885)	*P*^a^
Total no. of antenatal visits	48 030	18 425	21 507	38 377	NA
No. of antenatal intervention visits per pregnancy, median (IQR)	8.0 (3.0–12.0)	4.0 (2.0–6.0)	2.0 (1.0–4.0)	3.0 (2.0–5.0)	< 0.001
Gestational age at first intervention visit, median weeks (IQR)	13.4 (9.5–20.1)	27.1 (22.4–32.6)	27.7 (22.1–33.1)	21.9 (16.4–28.4)	< 0.001
First intervention visit at < 20 weeks gestation, no. (%) of pregnancies	4 523 (73.9)	638 (15.1)	1 141 (16.3)	4 432 (40.7)	< 0.001
First intervention visit at ≥ 20 weeks gestation, no.(%) of pregnancies	1 539 (25.1)	3 553 (83.9)	5 703 (81.4)	6 413 (58.9)	< 0.001
Gestational age uncertain, no. (%) of pregnancies	58 (0.9)	43 (1.0)	160 (2.3)	40 (0.4)	NA
Proteinuria measured at first intervention visit, no. (%) of pregnancies	5 676 (92.8)	4 143 (97.9)	6 372 (91.0)	10 769 (98.9)	NA
Proteinuria measured at subsequent intervention antenatal visits for hypertension, no. (%) of visits with hypertension	373/409 (91.2)	107/113 (94.7)	175/217 (80.7)	235/243 (96.7)	NA
Type of proteinuria dipstick used^b^	Mission® Urinalysis strips	Urine InstaTest strips	Medi-Test Protein 2 strips	Uristix® strips	NA

IQR: interquartile range; NA: not applicable.

^a^ *P*-values were based on comparisons of all groups by the *χ^2^*-test.

^b^ Mission® Urinalysis strips, ACON Laboratories, San Diego, United States of America; Urine InstaTest strips, Cortez Diagnostics, Woodland Hills, USA; Medi-Test Protein 2 strips, BHR Pharmaceuticals, Nuneaton, United Kingdom of Great Britain and Northern Ireland; Uristix® strips, Siemens, Erlangen, Germany.

Notes: *n* is the total number of pregnancies included in this analysis. At intervention visits women received a clinical assessment by a community health worker guided by a mobile health application. The trial protocol specified that proteinuria should be measured at the first intervention visit, and then at subsequent visits at which the woman was hypertensive. In Nigeria, proteinuria was measured at many subsequent visits regardless of blood pressure status (12 796/21 354 pregnancies, 59.9%).

At the first intervention visit, dipstick proteinuria was 1+ or above in less than 5% of pregnancies in all countries (India: 234/6120, 3.8%; Mozambique: 94/4234, 2.2%; Nigeria 286/7004, 4.1%; Pakistan: 315/10 885, 2.9%; Table 3). The prevalence of proteinuria ≥ 1+ was significantly different across countries (*P* < 0.001); it was highest in Nigeria, followed by India and then Pakistan, with the lowest prevalence in Mozambique. The ratio of pregnancies with 1+ proteinuria (as opposed to ≥ 2+) varied substantially from 5:1 in Pakistan to 1:1 in India (Table 3). However, in sensitivity analyses, adjustment for maternal characteristics and gestational age at the first intervention visit revealed that all countries had a lower prevalence of baseline proteinuria compared with India (Table 3).

**Table 3:** Prevalence of proteinuria at the first antenatal intervention visit in the Community-Level Interventions for Pre-eclampsia trial and relationship with adverse outcomes, 2013–2017

Variable	No. (%) of pregnancies				*P*
	India (*n* = 6 120)	Mozambique (*n* = 4 234)	Nigeria (*n* = 7 004)	Pakistan (*n* = 10 885)	
**Proteinuria **					
Total ≥ 1+	234 (3.8)	94 (2.2)	286 (4.1)	315 (2.9)	< 0.001^a^
1+	120 (2.0)	53 (1.3)	211 (3.0)	258 (2.4)	
≥ 2+	114 (1.9)	41 (1.0)	75 (1.1)	57 (0.5)	
Negative or trace	5 442 (88.9)	4 049 (95.6)	6 086 (86.9)	10 454 (96.0)	
Not assessed at first visit	444 (7.3)	91 (2.2)	632 (9.0)	116 (1.1)	
**Blood pressure measurements**					
No. of pregnancies with blood pressure measured	234	94	286	315	NA
Total with hypertension (%)	7 (3.0)	1 (1.1)	42 (14.7)	50 (15.9)	< 0.001
Proteinuria 1+	4	1	16	31	
Proteinuria ≥ 2+	3	0	27	19	
Total with normotension (%)	225 (96.2)	93 (98.9)	241 (84.3)	264 (83.8)	< 0.001
Proteinuria 1+	115	52	194	227	
Proteinuria ≥ 2+	110	41	47	37	
Not measured (%)	2 (0.9)	0 (0.0)	3 (1.0)	1 (0.3)	

NA: not applicable.

^a^ Our sensitivity analysis after adjusting for maternal characteristics revealed lower incidence of proteinuria ≥ 1+ when adjusted for age, parity and gestational age at first intervention visit (odds ratio, OR: 0.54; 95% confidence interval, CI: 0.45–0.66) in Pakistan; OR: 0.41 (95% CI: 0.32–0.54) Mozambique; and (OR: 0.76 (95% CI: 0.61–0.94) Nigeria) or with addition of level of education available in Mozambique (OR: 0.41; 95% CI: 0.31–0.54) and Pakistan (OR: 0.53; 95% CI: 0.43–0.66).

Notes: *n* is the total number of pregnancies included in this analysis. Inconsistencies arise in some values due to rounding.

In most of the pregnancies with proteinuria, the women were normotensive at the first intervention visit in each of the four countries (India: 225/234; Mozambique: 93/94; Nigeria: 241/286; Pakistan: 264/315; Table 3). Among women who were normotensive at their first visit, those with proteinuria had similar pregnancy outcomes, progression to hypertension, and maternal and perinatal mortality and morbidity compared with those without proteinuria (Table 4 and data repository).^6^ While the 95% CIs around the OR for these events were wide, the percentages were similar, with no consistent patterns of increasing proportions of pregnancies with adverse outcomes with increasing proteinuria (data repository).^6^ The results were similar when women with multiple pregnancies or those known to be HIV-positive were excluded in sensitivity analyses (data repository).^6^

**Table 4:** Relationship between proteinuria and adverse outcomes for 21 239 women with proteinuria without hypertension at their first intervention antenatal visit in the Community-Level Interventions for Pre-eclampsia trial, 2013–2017

Variable	No. (%) of pregnancies			OR (95% CI)	
	No proteinuria (*n* = 19 556)	Proteinuria ≥ 1+ (*n* = 394)	Proteinuria ≥ 2+ (*n* = 188)	Proteinuria defined as ≥ 1+	Proteinuria defined as ≥ 2+
**Progression to hypertension**	1 654 (8.5)	37 (9.4)	22 (11.7)	1.37 (0.53–3.51)	1.19 (0.3– 4.78)
**Maternal death or morbidity^a^**	1 862 (9.5)	61 (15.5)	15 (8.0)	0.98 (0.38–2.55)	0.88 (0.12–6.34)
**Death**	1 845 (9.4)	59 (15.0)	14 (7.5)	NA	NA
**Morbidity**	36 (0.2)	2 (0.5)	1 (0.5)	0.98 (0.38–2.55)	0.88 (0.12–6.34)
**Birth at < 37 weeks**	4 882 (25.0)	88 (22.3)	47 (25.0)	1.10 (0.58–2.07)	1.36 (0.4– 4.59)
**Caesarean delivery**	3 186 (16.3)	60 (15.2)	25 (13.3)	0.77 (0.36–1.65)	0.72 (0.19–2.78)
**Perinatal or neonatal morbidity**	3 164 (16.2)	73 (18.5)	30 (16.0)	0.86 (0.45–1.66)	1.01 (0.28–3.63)
**Perinatal mortality**	1 505 (7.7)	26 (6.6)	16 (8.5)	0.95 (0.32–2.82)	2.02 (0.22–18.44)
Stillbirth	729 (3.7)	12 (3.1)	10 (5.3)	0.99 (0.23–4.26)	2.95 (0.09–102.31)
Early neonatal death	639 (3.3)	10 (2.5)	5 (2.7)	0.70 (0.12–4.28)	1.07 (0.04–28.98)
**Neonatal morbidity**	2 001 (10.2)	54 (13.7)	16 (8.5)	0.80 (0.36–1.76)	0.63 (0.13– 3.03)

CI: confidence interval; NA: not applicable; OR: odds ratio.

**^a^** Morbidity included eclampsia and pulmonary oedema.

Notes: *n* is the total number of pregnancies included in the analysis. These analyses reflect data from India (6120 women), Pakistan (10 885 women) and Mozambique (4234 women), as trial surveillance data were not available in Nigeria.

We estimated that at the national level in the trial countries, there would be large numbers of antenatal care visits at which proteinuria testing would not be undertaken if restricted to women with hypertension (annually number of visits in India: 173 872 507; Mozambique: 7 739 561; Nigeria: 52 655 379; Pakistan: 43 143 051). The cost of supplies for a proteinuria assessment in the trial was approximately US$ 1 in each country (India: US$ 0.91; Mozambique: US$ 1.17; Nigeria: US$ 1.01; Pakistan: US$ 0.90; Table 5). We projected large annual cost savings by screening for proteinuria only when hypertension was found (India: US$ 153 223 981; Mozambique: US$ 9 055 286; Nigeria: US$ 53 181 933; Pakistan: US$ 38 828 746). In a sensitivity analysis based on a four-visit antenatal care model, projected cost savings were still substantial: India: US$ 86 936 253 visits annually; Mozambique: US$ 3 869 781; Nigeria: US$ 26 327 690; Pakistan: US$ 21 571 525.

**Table 5:** Costs of supplies used for proteinuria assessment in the Community-Level Interventions for Pre-eclampsia trial, 2013–2017

Item	India	Mozambique	Nigeria	Pakistan
**Urinary dipsticks**				
Cost of 100 dipsticks, US$^a^	13.23	37.50	22.40	11.84
Cost of 100 dipsticks, 2019 US$	14.44	40.27	24.44	12.92
Cost/dipstick, 2019 US$	0.14	0.40	0.24	0.13
**Testing cups**				
Cost of 500 cups, US$	219.38	219.38	219.38	219.38
Cost of 500 cups, 2019 US$	235.57	235.57	235.57	235.57
Cost per cup, 2019 US$	0.47	0.47	0.47	0.47
**Gloves**				
Cost of 100 gloves, US$	13.78	13.78	13.78	13.78
Cost of 100 gloves, 2019 US$	14.80	14.80	14.80	14.80
Cost per pair of gloves, 2019 US$	0.30	0.30	0.30	0.30
Cost per proteinuria assessment, 2019 US$	0.91	1.17	1.01	0.90

US$: United States dollar.

^a^ Table 2 shows the type of dipstick used in each country.

Notes: Dipsticks, cups and gloves were purchased in 2013 in India, Pakistan and Nigeria and 2014 in Mozambique. We calculated the costs in US$ for 2019 using calculator available at: https://www.usinflationcalculator.com/

## Discussion

In almost 30 000 pregnancies from 27 intervention clusters in sub-Saharan Africa and South Asia, we demonstrated a very low prevalence of dipstick proteinuria by visual assessment soon after antenatal care booking. The prevalence of proteinuria in each country was related to maternal characteristics and gestational age at booking. Few women with baseline proteinuria had hypertension at the first intervention visit and among those with blood pressure in the normal range, there was no compelling relationship between baseline proteinuria and adverse pregnancy outcomes.

Previous studies of proteinuria screening have focused on its role in pre-eclampsia diagnosis in women with hypertension, rather than the added value of proteinuria screening when blood pressure is normal. Even WHO regards routine baseline assessment and ongoing surveillance of proteinuria testing as good practice without the need for evidence review.^2^ Our findings suggest otherwise.

Our measure of baseline proteinuria may reflect chronic kidney disease, pre-eclampsia (for women assessed at gestational age ≥ 20 weeks) or another process, such as dehydration, vaginal contamination, urinary tract infection, exercise or orthostatic proteinuria. Chronic kidney disease complicates up to 3% of pregnancies.^17^^,^^18^ Our results are consistent with this estimate, although we have inferred the possibility of chronic kidney disease from baseline proteinuria, rather than the accepted diagnostic tests in pregnancy that are not performed routinely anywhere (serum creatinine, quantitative proteinuria evaluation and urinalysis). Estimated glomerular filtration rate is inaccurate in pregnancy.^19^^,^^20^ Although chronic kidney disease in pregnancy is mostly mild, it is still associated with adverse pregnancy outcomes.^21^ As we were unable to demonstrate a relationship between baseline dipstick proteinuria and adverse pregnancy outcomes among almost 30 000 pregnancies, it may be that the relationship between chronic kidney disease and adverse outcomes is different in our study settings. More likely, dipstick proteinuria has such low diagnostic accuracy for true proteinuria and underlying chronic kidney disease that it is not a useful test.^2^

We were unable to estimate the incidence of new proteinuria without hypertension, which has been documented by others to be low (0.5% to 1.9%) in unselected pregnancies.^3^^,^^22^ Most of these women (75.3%) did not develop hypertension indicative of pre-eclampsia. In our trials, the number-needed-to-screen by blood pressure measurement to detect pregnancy hypertension is 10 women (80 visits). In contrast, the number-needed-to-screen by dipstick proteinuria to detect proteinuria that will progress to pre-eclampsia is at least 213–769 women (1704–6152 visits) as these women will of course also be screened with blood pressure measurement. These calculations are based on an incidence of pregnancy hypertension of about 10%,^14^ gestational proteinuria of 0.5–1.9% in unselected pregnancies,^3^^,^^22^ progression of gestational proteinuria to pre-eclampsia of 24.7% of pregnancies,^3^^,^^22^ and an eight-contact antenatal care model^2^ in which all women undergo blood pressure measurement and proteinuria screening at each contact.

We believe the practice of proteinuria screening among normotensive women in pregnancy should be questioned. First, there is the high volume and cost of testing to detect one woman with isolated proteinuria and normal blood pressure. Second, our findings suggest there is no compelling relationship between isolated baseline proteinuria and adverse outcomes. Third, after the booking visit, there is no evidence that subsequent antenatal care contacts with proteinuria testing (versus those without) result in superior outcomes. Finally, more than 80% of proteinuria occurs in the third trimester.^23^

We have demonstrated that screening for proteinuria only in the presence of hypertension could be undertaken to inform a diagnosis of pre-eclampsia. This practice would not increase the incidence of pre-eclampsia, as most women in our trials presented with non-severe, gestational hypertension.^14^ Importantly, however, this practice could be associated with large cost savings for health systems in low-resource settings.^14^ Although the cost of each proteinuria screen is low (about US$ 1), the use of dipstick screening at each antenatal care contact for each woman results in a large cumulative sum. In line with the Choosing Wisely movement, it is reasonable to ask whether those funds could be used in other ways to optimize outcomes.^4^

The strengths of our study include evaluation of a large number of women in four low-resource sub-Saharan and south Asian countries. We estimated baseline proteinuria prevalence independent of hypertension, and considered the utility of this measurement using common definitions of dipstick proteinuria. 

Some limitations of our study include having proteinuria measurements from most but not all women, from bookings only in community care and not at subsequent antenatal care visits by women with normotension, and from measurements by CHWs. We do not know how many women initially tested negative for proteinuria but later developed isolated proteinuria. However, it is unlikely that we missed earlier presentations of pre-eclampsia, as the incidence of gestational hypertension in our trials (6.5–8.4%)^14^ was as high as in settings where antenatal care contacts are frequent. Second, we had data only on basic maternal characteristics for adjusted analysis of proteinuria prevalence. As is typical in the settings where we carried out the trials, no reliable information was available on women’s past history of chronic hypertension or renal disease to differentiate prior renal disease from pre-eclampsia (if booking occurred after 20 weeks). We had no information on the type of antiretroviral therapy taken by women with HIV (although exclusion of HIV-positive women left the results unchanged). We also had no direct measure of dehydration or any information on whether women were dehydrated due to manual labour occupations or a lack of toilet facilities. Third, while the 95% CIs were wide around our outcome estimates, we must question the importance of an effect that cannot be demonstrated among more than 30 000 women. The relationship between baseline proteinuria and adverse outcomes would only have been strengthened by the fact that more than 50% of women in Pakistan and most women in Mozambique booked after 20 weeks, as their baseline proteinuria could have reflected pre-eclampsia. Finally, we illustrated potential health system cost savings of a strategy of proteinuria testing only in pregnant women with hypertension. However, we did not undertake a formal cost–consequences analysis and we acknowledge that government bulk purchase of testing supplies may lower costs. Also, while our calculations were based on an eight-visit antenatal care model, we estimate that a four-visit model would still be associated with substantial numbers of proteinuria assessments avoided in normotensive pregnancy and cost savings.

In conclusion, our findings do not support the usefulness of proteinuria screening at the first assessment in pregnancy. This practice should be re-evaluated and robust health economic studies undertaken, to avoid unnecessary tests and treatments that fail to add value to care, consume resources and may cause harm through follow-up investigation and worry for women.^4^
